# Increased Risk of Hypothyroidism in People with Asthma: Evidence from a Real-World Population-Based Study

**DOI:** 10.3390/jcm11102776

**Published:** 2022-05-14

**Authors:** Shih-Cheng Huang, Shuo-Yan Gau, Jing-Yang Huang, Wen-Jun Wu, James Cheng-Chung Wei

**Affiliations:** 1 Institute of Medicine, Chung Shan Medical University, Taichung City 40201, Taiwan; huangesir@gmail.com (S.-C.H.); wchinyang@gmail.com (J.-Y.H.); 2 School of Medicine, Chung Shan Medical University, Taichung City 40201, Taiwan; sixsamurai.shien15@gmail.com; 3 Center for Health Data Science, Chung Shan Medical University Hospital, Taichung City 40201, Taiwan; 4 Department of Microbiology and Immunology, School of Medicine, Chung Shan Medical University, Taichung City 40201, Taiwan; 5 Department of Allergy, Immunology and Rheumatology, Chung Shan Medical University Hospital, Taichung City 40201, Taiwan; 6 Graduate Institute of Integrated Medicine, China Medical University, Taichung City 40402, Taiwan

**Keywords:** hypothyroidism, asthma, real-world study, cohort study

## Abstract

Background: Non-T2 asthma and hypothyroidism share several inflammatory mechanisms in common. However, large-scale, real-world studies evaluating the association between asthma and hypothyroidism are lacking. The objective of this study was to evaluate the risk for asthma patients of developing hypothyroidism. Methods: In the retrospective cohort study, people with asthma were recruited from the Longitudinal Health Insurance Database in Taiwan. After excluding ineligible patients with a previous history of hypothyroidism, 1:1 propensity matching was conducted to select a non-asthma control group. Based on the multivariate Cox regression model, the adjusted hazard ratio of asthma patients developing hypothyroidism was calculated. Results: In total, 95,321 asthma patients were selected as the asthma group and the same amount of people without asthma were selected as the control group. The incidence levels of new-onset hypothyroidism in asthma and non-asthma groups were 8.13 and 6.83 per 100,000 people per year, respectively. Compared with the non-asthma group, the adjusted hazard ratio of the asthma group developing hypothyroidism was 1.217 (95% confidence interval, 1.091–1.357). Conclusions: We found having asthma to be associated with an increased risk of hypothyroidism. Clinicians should be concerned regarding the endocrinological and inflammatory interaction between the two diseases while caring for people with asthma.

## 1. Introduction

Because its presence indicates the providing of insufficient thyroid hormones for tissues and body organs, hypothyroidism is regarded as one of the critical endocrinological diseases which could potentially cause great impairment to a patients’ quality of life [1]. Risk factors for hypothyroidism include a history of autoimmune-related diseases such as type1 diabetes [2] and celiac diseases [3]. Hashimoto’s thyroiditis is one of the common sub-types of autoimmune-related hypothyroidism. In recent years, the pathogenesis of Hashimoto’s thyroiditis has been thought to be highly involved in Th1 and Th17 inflammatory pathways [4,5].

As an inflammatory respiratory disease, asthma is related to symptoms including coughing, wheezing and shortness of breath [6]. The prevalence of asthma varies widely based on the geographical location. According to a recent meta-analysis conducted in Iran, the prevalence of asthma was estimated to be 6% in children and 8% in grown adults, respectively [7]. An epidemiological report in Taiwan reported that the prevalence of asthma was 11.9% in the Taiwanese population [8]. The mechanism involved in asthma pathogenesis has long been regarded as T2-mediated [9]. However, increasing evidence indicates that non-T2 asthma, which refers to asthma involved in the Th1 or Th17 pathway instead of the Th2, could also play a substantial role in the related inflammatory mechanisms [10].

Given that some of the mechanisms such as the Th17 pathway show an overlap between autoimmune-related hypothyroidism and non-T2 asthma, associations could potentially exist between the two diseases. Previous studies have indicated that thyroid disorders could be non-respiratory comorbidities of asthma [11]. Based on their similar autoimmune etiology, hyperthyroidism and hypothyroidism-related autoantibodies have been found to be highly present in asthma patients [11,12]. A recent population-based real-world cohort study has demonstrated the association between asthma and new-onset hyperthyroidism [13]. However, large-scale studies evaluating the risk of asthma patients developing hypothyroidism are lacking. To clarify the nature of the interactions between asthma, thyroid function and the endocrine system, we conducted a retrospective population-based cohort study to evaluate the asthma–hypothyroidism association based on the same dataset as a previously published study evaluating the association between asthma and hyperthyroidism [13].

## 2. Materials and Methods

### 2.1. Data Source

The Longitudinal Health Insurance Database (LHID), a sub-dataset from the Taiwanese National Health Insurance Research Database (NHIRD), has been utilized in the current study. The NHIRD is a database based on a compulsory National Health Insurance program in Taiwan, which has a greater than 90% coverage and has been widely utilized in previous studies [14,15,16]. Information regarding patients’ hospitalization, diagnostic records, urbanization and income data were available in the LHID. Disease diagnoses were recorded based on the International Classification of Disease, Ninth. Revision, Clinical Modification (ICD-9-CM). In the NHIRD, all enrolled participants were deidentified because personal information was not available. Thereby, we were exempted from the requirement to gain patients’ consent as it was not applicable.

### 2.2. Study Population

**Figure 1:**
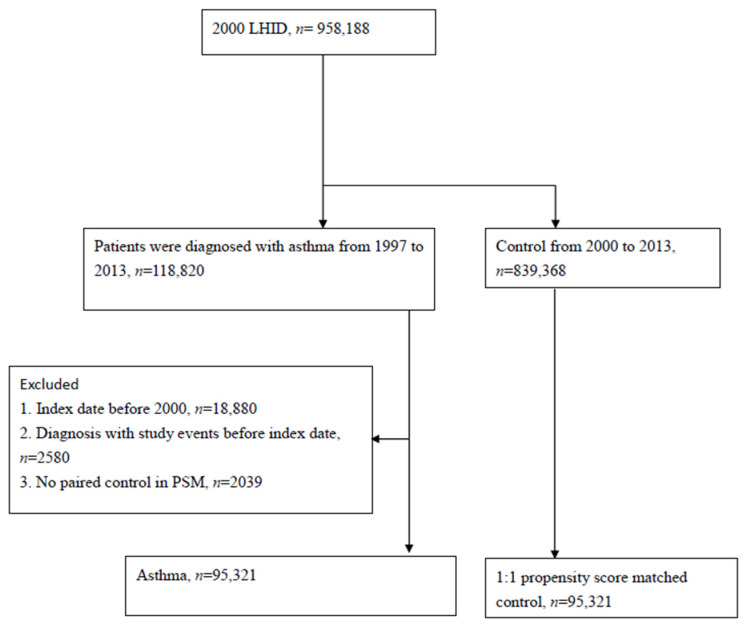
Study flow chart of patient selection.

Between 1997 and 2013, patients with asthma were enrolled in the study design. The definitions of the asthma groups were based on the records of asthma diagnoses (ICD-9-CM code 493). Based on a 1:1 propensity score matching, people with no asthma were chosen as a control (non-asthma) group. The propensity score was determined based on the study participants’ baseline information, including urbanization status, income information, medical utilization status and comorbidities (including Esophageal reflux, Hypertension, Coronary artery disease, Diabetes mellitus, Hyperlipidemia, Systemic Lupus Erythematosus, Sjogren syndrome, Chronic kidney disease, Chronic liver diseases, Gastritis and duodenitis, Malignancies and Depression). People meeting the following criteria were excluded from the study design: (1) people with an index date before 2000; (2) people with no appropriate paired control in the propensity score matching; (3) people having a previous record of thyroid dysfunction before the index date. Detailed information regarding patient selection is available in Figure 1.

### 2.3. Outcome Measurement and Covariates

The main outcome of the current study was the incidence of new-onset hypothyroidism. The occurrence of hypothyroidism was identified based on the diagnoses records of hypothyroidism (ICD-9-CM 244). To enhance the validity of hypothyroidism diagnosis, only subjects with more than two out-patient visits or one hospitalization record would be regarded as having incident hypothyroidism.

### 2.4. Statistical Analysis

For data analyses, SAS (version 9.4; SAS Institute, Cary, NC, USA) software was utilized. The PSM was conducted for the purpose of balancing the baseline information for asthma and non-asthma groups. The PSMATCH procedure in the SAS software was conducted based on “greedy nearest neighbor matching”. In the current study, covariates, including age, gender, urbanization status, medical resources utilization status and related comorbidities (Esophageal reflux, Hypertension, Coronary artery disease, Diabetes mellitus, Hyperlipidemia, Systemic Lupus Erythematosus, Sjogren syndrome, Chronic kidney disease, Chronic liver diseases, Gastritis and duodenitis, Malignancies and Depression), were considered. The standardized differences were calculated to determine the balanced difference between the asthma and non-asthma groups. With the value of the standardized difference being less than 0.10, the comparison between the groups could be viewed as showing no difference. For the evaluation of hypothyroidism risk and incidence rate, a crude and adjusted hazard ratio was applied to multivariate Cox regression analyses. The cumulative incidence of the outcome event was determined based on a Kaplan–Meier plot. A 95% confidence interval and log-p rank test were applied to determine the statistical significance. With a *p* value < 0.05, the statistic test can be regarded as statistically significant.

## 3. Results

From the 958,188 participants in the LHID, there were eventually 95,321 eligible people with asthma selected as the asthma group after excluding 20,499 patients meeting the exclusion criteria (Figure 1). Before the propensity matching, differences existed in the medical utilization statuses (length of hospital stay before the index date) and specific comorbidities (Esophageal reflux, Hypertension, Coronary artery disease, Hyperlipidemia, Chronic liver diseases and Gastritis and duodenitis). After 1:1 propensity matching, the considered covariates in this study, including age, sex, comorbidities, and medical utilization, were of insignificant difference (Table 1). The median time of follow-up for the asthma group was 97 months, and the median follow-up time for the non-asthma group was the same. In both the asthma and non-asthma groups, the percentage of males was 51% and the percentage of females was 49%. Additionally, most of the participants lived in an urban area.

Table 2 presented study groups for the incidence of hypothyroidism. In the non-asthma group, the incidence rate for new-onset hypothyroidism was 6.83 per 100,000 person-months (95% CI, 6.31–7.40). Whereas for people with asthma, the incidence rate of hypothyroidism was 8.13 (95% CI, 7.55–8.75). The risk for the asthma group to develop incident hypothyroidism was 18.8% higher than for people without asthma (crude hazard ratio = 1.18, 95% CI, 1.066–1.325).

**Figure 2:**
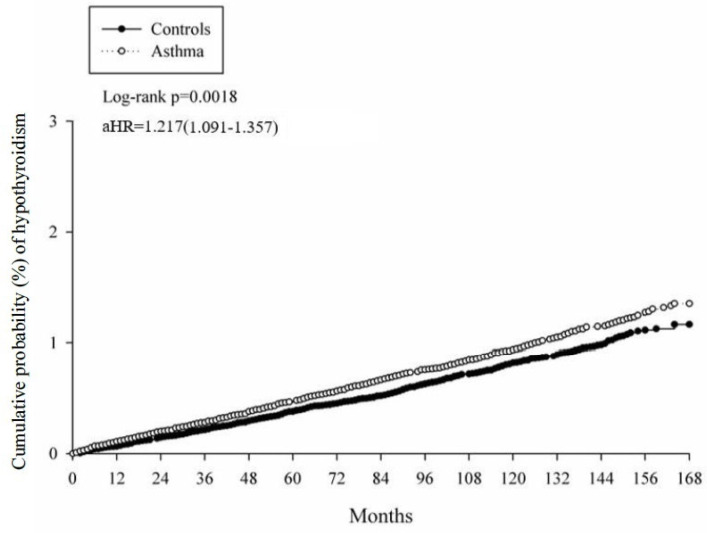
The cumulative incidence curves of developing hypothyroidism for patients with and without asthma.

Table 3 presents the results from the multivariate Cox proportional hazard regression for the evaluation of the adjusted hazard ratio (aHR) for hypothyroidism in asthma and non-asthma groups. Using a multivariate model considering related covariates, having asthma was shown to be associated with a higher risk of developing hypothyroidism, with an increased risk of 21.7% compared to those without asthma (aHR = 1.217, 95% CI, 1.091–1.357). The cumulative incidence was evaluated in the Kaplan–Meier plot shown in Figure 2. Comorbidities could serve as potential risk factors for developing hypothyroidism. For instance, the presence of Sjogren syndrome was associated with an aHR of 1.776 (95% CI, 1.136–2.777) for future hypothyroidism development and the presence of chronic kidney diseases were associated with a more than 80% higher risk of developing new-onset hypothyroidism (aHR = 1.816, 95% CI, 1.333–2.474).

## 4. Discussion

The current population-based cohort study provided real-world evidence regarding the association between asthma and hypothyroidism. Having asthma was associated with an increased risk of hypothyroidism, with an aHR of 1.217 after considering related confounders.

An association between hypothyroidism and respiratory diseases has been discussed in previous studies. In a previous case-control study, Adegunsoye et al. stated that the presence of autoimmune hypothyroidism was associated with chronic hypersensitivity pneumonitis [17]. The influence on thyroid function was reported to serve as a potential indicator of chronic hypersensitivity pneumonitis mortality [17]. Coexistences of hypothyroidism and asthma have also been reported in case reports [18,19]. In a recent cohort study, Liu et al. reported that maternal hypothyroidism could serve as a risk factor for asthma incidence in offspring [20]. According to previous studies, medications for asthma treatment, such as corticosteroids, though able to potentially influence the concentration level of thyroid-stimulating hormones, do not significantly increase the risk of clinical new-onset hypothyroidism [21]. Common causes of secondary drug-induced hypothyroidism include the utilization of iodine-containing drugs, such as amiodarone, iodoquinol or idoxuridine, which are less utilized in asthma patients [22]. Therefore, confounders caused by comedications did not seem to greatly influence the observed association. Though the current available evidence was insufficient to determine the actual mechanism regarding the immunological and endocrinological interactions between hypothyroidism and asthma, our study could provide credible evidence for the asthma–hypothyroidism association.

Though asthma is generally viewed as involved in Th2-related inflammation, non-T2 asthma has been identified in order to explain the alternative pathway of different Th2-mediated mechanisms in recent years [23]. Known also as “non-eosinophilic asthma”, non-T2 asthma presented mostly with neutrophil instead of eosinophil in the blood or sputum of patients [23,24]. Instead of T2-related cytokines, cells, such as Th1 and Th17, and downstream cytokines, such as IL-6 and IL-17, were involved in the mechanism of non-T2 asthma, leading to subsequent airway impairment [24,25,26]. Likewise, mechanisms including Th1 and Th17 in the inflammatory pathway were reported to be involved in the pathogenesis of hypothyroidism [1]. For people with severe autoimmune hypothyroidism, the Th1-related cells such as interferon gamma were secreted at a higher level compared to in those with mild autoimmune hypothyroidism [27]. Likewise, the Th1/Th2 ratio was observed to increase in people with severe autoimmune hypothyroidism [27]. As for Th17 involvement, the Th17 cytokine level was found to be higher in people with hypothyroidism than in people who did not have hypothyroidism [27,28]. In HCV-infected patients, the dysregulation of Th17 cytokines was reported to be associated with the subsequent presence of hypothyroidism [29]. Likewise, in the pregnant population, imbalanced IL-17 levels could serve as a risk factor for subclinical hypothyroidism [30]. According to current evidence, the Th17 family could play a potentially critical role in the development of hypothyroidism. Given that non-T2 asthma is significantly involved in the Th17 pathway, the observed association in this study could possibly be explained. Moreover, since in a previous population-based study, new-onset hyperthyroidism was reported to be associated with asthma [13], it is possible that an interplay between asthma and autoimmune-related thyroid diseases could exist. Though hyperthyroidism and hypothyroidism clinically showed different presences of thyroid function, the pathogenesis of Graves’ Disease and Hashimoto thyroiditis were both reported to be associated with the Th-17 pathway and the IL-17 family cytokines [28,31,32]. Given that the Th-17 pathway was critical in the mechanism of non-T2 asthma, it is possible that the Th-17 pathway mediates the interaction between asthma and thyroid dysfunction. Future studies are recommended that focus on this influence in actual lab data (for instance, the concentration of free T3/T4) in asthma patients in order to evaluate the interactions between non-T2 asthma, hyperthyroidism and hypothyroidism.

The strength of this study is based on the robust population-based dataset it utilized. Through the randomized and de-identified data retrieved from the NHIRD and the LHID, the issues of selection bias and recalling bias could also be addressed to a great degree. However, there were several limitations that should be stated. First, residual confounders could exist. Factors such as BMI status may influence the onset of hypothyroidism [33] and hormone secretion status may influence the pathogenesis of asthma [34]. Nonetheless, due to the limitations of the NHIRD, information regarding BMI, lifestyle, smoking status and precise lab data for thyroid hormones were not available. In this case, confounding biases caused by residual confounders could exist. Nevertheless, we did try our best to address possible confounding biases. In the current analysis model, the related comorbidities were adjusted as potential confounders to cope with the potential influence of confounders. Second, medical utilization status could also lead to potential monitoring bias. Compared to those in the health control group, people with asthma may have a higher tendency to go to medical institutions and take the related examinations. In this case, the tendency to be diagnosed with hypothyroidism for people in the asthma group might be higher than for the healthy control group because of their increased frequency of medical utilization. To address this limitation, we included the length of hospital stays as one of the covariates to adjust for the difference in medical utilizations. At the baseline, a difference in medical utilization was not statistically significant after matching was performed. Third, the issue of the specific immunophenotype our recruited patients could be a potential limitation in our study. The identification of diseases in the Taiwanese NHIRD were based on the diagnostic code of ICD-9, which was created by specialists. However, due to its administrative intentions, the code might not be enough to identify diseases [35]. In the previous work published from the LHID, the definitions of the asthma cohort included the prescriptions for related medications [13]. Nonetheless, in the current study’s design, we only utilized the ICD diagnosis as the definition of asthma in order to make the population we chose to include avoid potential selection bias. Setting medication use (i.e., inhaled corticosteroid) as an additional inclusion criterion could potentially lead to selection bias. Given that corticosteroid use may possibly influence endocrinological and infection status [36], those with a chronic utilization pattern for these kinds of medications could potentially have a higher tendency to take endocrinological examinations. In this case, the possibility of medication users being diagnosed with endocrinological diseases could be misinterpreted. To the best of our knowledge, a validation study regarding the definition of asthma in the ICD-9 diagnostic system is lacking. Hence, the current definition we adopted for this study has also been utilized in the previous asthma-related literature that is based on the NHIRD [37]. Moreover, given that T2/non-T2 asthma does not have a specific code in the ICD-9 system, we were not able to clearly identify the phenotype via the diagnostic codes. Since the NHIRD was not able to provide detailed information regarding patients’ lab data, the distributions and statuses of T2/non-T2 asthma in our patients were also unavailable. Hence, additional analyses evaluating non-T2 asthma and its association with new-onset hypothyroidism could not be conducted to further demonstrate our hypothesis in this discussion. To address the issue, we only included those patients with more than two out-patient visits or one inpatient visit for hypothyroidism as our outcome event to validate the definition. The applied codes for diseases of interest and related comorbidities have also been utilized in previous real-world studies [15,38,39].

In conclusion, the current population-based cohort study reports an increased risk of hypothyroidism in people with asthma. Clinicians should be concerned regarding the endocrinological and inflammatory interaction between the two diseases while caring for people with asthma. Future studies are recommended which focus on the actual mechanism and interaction between asthma and thyroid dysfunction.

## Figures and Tables

**Table 1:** Baseline characteristics among the study groups.

	Before PSM ^1^ (1:4 Age-Sex Matching)			After PSM		
	Control *n* = 192,686	Asthma *n* = 96,343	Standardized Difference	Control *n* = 95,321	Asthma *n* = 95,321	Standardized Difference
Sex			0.000			0.011
Female	98,494 (51.12%)	49,247 (51.12%)		48,789 (51.18%)	48,716 (51.11%)	
Male	94,192 (48.88%)	47,096 (48.88%)		46,532 (48.82%)	46,605 (48.89%)	
Age			0.000			0.028
<30	68,672 (35.64%)	34,336 (35.64%)		33,765 (35.42%)	34,282 (35.96%)	
30–45	31,428 (16.31%)	15,714 (16.31%)		15,024 (15.76%)	15,561 (16.32%)	
45–65	47,734 (24.77%)	23,867 (24.77%)		23,439 (24.59%)	23,421 (24.57%)	
≥65	44,852 (23.28%)	22,426 (23.28%)		23,093 (24.23%)	22,057 (23.14%)	
Urbanization			0.019			0.009
Urban	113,343 (58.82%)	56,663 (58.81%)		56,502 (59.28%)	56,156 (58.91%)	
Sub-urban	58,398 (30.31%)	28,691 (29.78%)		28,282 (29.67%)	28,387 (29.78%)	
Rural	20,945 (10.87%)	10,989 (11.41%)		10,537 (11.05%)	10,778 (11.31%)	
Low income	1085 (0.56%)	804 (0.83%)	0.033	653 (0.69%)	760 (0.8%)	0.013
Length of hospital stays (Within 2 year before index date)			0.248			0.010
0 day	164,761 (85.51%)	73,171 (75.95%)		72,752 (76.32%)	73,007 (76.59%)	
1–6 days	15,617 (8.10%)	11,795 (12.24%)		11,891 (12.47%)	11,579 (12.15%)	
≥7 days	12,308 (6.39%)	11,377 (11.81%)		10,678 (11.2%)	10,735 (11.26%)	
Comorbidity						
Esophageal reflux	3449 (1.79%)	3576 (3.71%)	0.118	3118 (3.27%)	3233 (3.39%)	0.007
Hypertension	36,107 (18.74%)	25,188 (26.14%)	0.178	25,168 (26.4%)	24,345 (25.54%)	0.020
Coronary artery disease	13,612 (7.06%)	11,946 (12.4%)	0.181	11,281 (11.83%)	11,244 (11.8%)	0.001
Diabetes mellitus	17,036 (8.84%)	10,597 (11%)	0.072	10,538 (11.06%)	10,267 (10.77%)	0.009
Hyperlipidemia	18,735 (9.72%)	12,887 (13.38%)	0.114	12,683 (13.31%)	12,380 (12.99%)	0.009
SLE ^1^	229 (0.12%)	209 (0.22%)	0.024	188 (0.20%)	192 (0.20%)	0.001
Sjogren syndrome	933 (0.48%)	653 (0.68%)	0.025	626 (0.66%)	617 (0.65%)	0.001
Chronic kidney disease	2357 (1.22%)	1626 (1.69%)	0.039	1571 (1.65%)	1552 (1.63%)	0.002
Chronic liver diseases	14,447 (7.5%)	11,333 (11.76%)	0.145	10,958 (11.5%)	10,756 (11.28%)	0.007
Gastritis and duodenitis	20,371 (10.57%)	16,968 (17.61%)	0.203	16,477 (17.29%)	16,122 (16.91%)	0.010
Malignancies	5467 (2.84%)	3501 (3.63%)	0.045	3593 (3.77%)	3381 (3.55%)	0.012
Depression	4469 (2.32%)	3779 (3.92%)	0.092	3498 (3.67%)	3502 (3.67%)	0.000

^1^ PSM, propensity score matching; SLE, systemic lupus erythematosus.

**Table 2:** Incidence of hypothyroidism in the PSM study group.

	Control *n* = 95,321	Asthma *n* = 95,321
Follow-up person-months	8,737,305	8,724,870
hypothyroidism	597	709
Incidence rate * (95% CI)	6.83 (6.31–7.40)	8.13 (7.55–8.75)
Crude Relative risk (95% CI)	Reference	1.188 (1.066–1.325)

* Incidence rate, per 100,000 person months.

**Table 3:** Multiple Cox proportional hazard regression for the estimation of adjusted hazard ratios on Hypothyroidism.

Variable	aHR (95% CI)
Asthma (ref: Control)	**1.217** (1.091–1.357)
Sex (ref: Female)	
Male	0.41 (0.362–0.464)
Age (ref: 30–45)	
<30	0.316 (0.255–0.392)
45–65	1.483 (1.247–1.765)
≥65	1.796 (1.481–2.178)
Urbanization (ref: Urban)	
Sub-urban	0.842 (0.743–0.955)
Rural	0.793 (0.664–0.948)
Length of hospital stays ^1^ (ref: 0 day)	
1–6 days	1.118 (0.953–1.311)
≥7 days	1.107 (0.933–1.312)
Comorbidity	
Esophageal reflux	1.347 (0.981–1.851)
Hypertension	1.066 (0.929–1.223)
Coronary artery disease	1.357 (1.174–1.568)
Diabetes mellitus	1.031 (0.879–1.209)
Hyperlipidemia	1.055 (0.91–1.223)
SLE ^2^	1.462 (0.691–3.097)
Sjogren syndrome	1.776 (1.136–2.777)
Chronic kidney disease	1.816 (1.333–2.474)
Chronic liver diseases	1.115 (0.962–1.292)
Gastritis and duodenitis	1.22 (1.074–1.386)
Malignancies	1.854 (1.509–2.278)
Depression	1.092 (0.867–1.375)

^1^ Length of hospital stays were the sum of the days in hospital within two years before the index date.^2^ SLE, systemic lupus erythematosus.

## Data Availability

The current study utilized datasets from the Longitudinal Health Insurance Database (LHID) 2000, which is a subset of the NHIRD. The dataset was administered by the Taiwan National Health Insurance (NHI) Bureau and was not made publicly available due to the Taiwanese “Personal Information Protection Act”. Formal requests may be submitted to the Taiwanese NHI for permission to disclose related data (https://dep.mohw.gov.tw/dos/cp-5146-59456-113.html, last accessed date on 10 May 2022).
